# Community-based urgent care in Israel and worldwide

**DOI:** 10.1186/2045-4015-2-38

**Published:** 2013-10-23

**Authors:** Deena R Zimmerman

**Affiliations:** 1 Terem Emergency Medical Centers, Rechov Yirmiyahu 80, Jerusalem, Israel

**Keywords:** (3–10) Urgent care, Outpatient health services, Health policy - Israel

## Abstract

**Background:**

Intermittent treatment of acute lower acuity situations has come to be defined as urgent rather than emergent care. The location of urgent care delivery has been shifting from exclusively hospital or office settings to other community locales.

**Aims:**

To review the concept of urgent care and the new models of health care delivery in the niche between hospitals and primary care. To highlight the roles of urgent care in Israel and compare these roles with those in other countries.

**Method:**

Narrative review of the literature.

**Main findings:**

The new models of community based urgent care include 1) the urgent care center; 2) the retail or convenience clinic, 3) the free standing emergency center, and 4) the walk-in clinic. These models fall on a continuum of comprehensiveness. They offer care at a lower cost than hospital-based emergency departments and greater temporal convenience than primary care physicians. However, their impact on emergency department utilization and overcrowding or primary care physician overload is unclear.

Israel has integrated its urgent care centers into its national health system by encouraging the use of urgent care centers and by requiring all health insurance funds to reimburse patients who use these centers. This integration is similar to the approach in England; however, the type of service is different in that the service in England is provided by nurses. It is different from most other countries where urgent care facilities are primarily private ventures.

**Conclusions:**

Community-based acute care facilities are becoming a part of the medical landscape in a number of countries. Still, they remain primarily on the fringe of organized medicine. Despite the important role of community-based acute care facilities in Israel, no nationwide study has been done in two decades. Health policy planning in Israel necessitates further study of urgent care use and its clinical outcomes.

## Introduction

From the middle of the 20^th^ century, the growing use of technology in medicine and the decreased willingness of physicians to make house calls [1] led to the provision of emergent care primarily by hospital based emergency departments (EDs). However, over the last four decades, there has arisen the concept of urgent as opposed to emergent care. “Urgent care” is commonly used to refer to intermittent health care offered in the community for acute lower acuity situations.

Obviously, many office-based visits to physicians could fit under this definition. However, office settings have delineated hours, while the need for treatment may arise outside of that framework. Furthermore, schedules may be filled in advance and frequently lack the flexibility to allow additional appointments. Thirdly, physicians’ offices often lack laboratory, radiological or treatment facilities on site. The term “urgent care” assumes greater temporal availability and more ancillary services than the traditional physician’s office.

Despite the proliferation of urgent care models world-wide, there has been no recent review of the overall concept and no comparison of Israeli and international models.

The goal of this paper is to provide a narrative review of the spectrum of urgent care models that exist worldwide and the health policy implications of this form of medical care. To achieve this goal, we searched Pubmed and Google Scholar using the key words urgent care, walk in clinics, retail clinics, free standing emergency facilities and free standing emergency departments to create a narrative review of the literature. In the sections that follow, we will describe the urgent care models and their evolution and compare and contrast the services they provide. Particular focus will be placed on current Israeli urgent care models. We then critically review the arguments that have been offered in favor of urgent care and conclude with unique issues related to urgent care in Israel.

### Urgent care models

A number of arguments been offered for the need for community based services that fill in the gap between the comprehensive services addressing acute problems provided by EDs and the ongoing care provided by physicians in traditional office settings. These arguments include reduction of ED utilization for minor complaints with its attendant costs, reduction of ED overcrowding, reduction of waiting time for outpatient physician appointments, and patient convenience. To reach these goals, four models have been developed to date. These include 1) the urgent care center; 2) the free standing emergency center; 3) the retail or convenience clinic; and 4) the walk in clinic. We will first outline the development of each model and then compare and contrast how each model attempts to meet these goals. It should be noted that the health care systems in which these models function vary from country to country.

#### Urgent care centers

This was the first model that was devised to close the gap between the office and the ED. It originated in the United States and was called a free-standing emergency center (FEC) [2]. The first such centers were privately owned and operated clinics founded independently in 1973 in Rhode Island and Delaware [3,4]. These facilities were envisioned as functioning as low-acuity emergency rooms and providing services when seeing one’s regular physician quickly was impossible or impractical. Initially, most of these clinics had expanded working hours (approximately 15 hours per day) but not 24- hour-per-day accessibility [5]. Alternate names have been used for this practice model such as emergicenters and urgycenters. The current widely used term is the urgent care center (UCC). Over 1,000 UCCs were in operation in 1983 in the United States and the current estimate is approximately 9,000 [6].

Urgent care centers that fit this model are becoming an important component outside of North America as well:

•New Zealand has had urgent care centers for since the late 1980s [7]. These clinics are run primarily as private ventures [8].

•In 2011, a number of urgent care centers were opened in Victoria, Australia. There is a move underway to replace the emergency department of Australia’s Bulli Hospital with an urgent care center and Health Direct Australia provides a number of overnight urgent services in the North Territory [9].

•In parts of Ireland, General Practitioners (GPs) have come together to form co-operatives providing medical service outside normal working hours. These “out of hour co-operatives” receive financial assistance from the state. The co-operative may be based in a health centre, public hospital or in another location and is usually staffed by participating GPs in a rotating fashion. Unlike the UCC models described above, the service is generally available only to the patients of the GPs participating in the co-op [10].

•Hungary and Bahrain are also listed has having facilities that are members of the Association of Urgent Care of America; however, we were not able to find any further details about the care in those countries [11].

The first UCC in Israel were opened in 1988. Prior to this point, the primary out-of-hours care location was the hospital ED as, in Israel, community physicians are only available during office hours and there is no concept of night call for these professionals. Another, very limited, option was first aid services provided in some branches of Magen David Adom, Israel’s equivalent of the Red Cross, in a government funded service known as Sharal (an acronym for Night Medical Services in Hebrew). This service ended in approximately 1996 [12].

Unlike the United States, where UCCs were opened at first by private physicians and this model was later followed by insurers, in Israel, insurers have been involved from the start. The first UCCs (called MARAM – an acronym of Location for Immediate Care in Hebrew) were founded by Clalit, the largest health insurer of the Israeli population. The health plan’s stated goal for UCC establishment was to provide a cheaper option than emergency rooms for out-of-hours care. The Clalit model involved extended hours of care (generally until midnight) at some of its clinics [12]. Currently, Clalit has 49 clinics that are listed on its website for provision of urgent care, not counting those where the service is provided by the private companies described below. All but one of these clinics are open on evenings and weekends. The clinic in Kfar Kasem is open 24 hours per day. Most of these clinics have physicians only. In 9 of the 49 clinics there are radiological services. Ten of the clinics provide basic laboratory services with an additional 29 providing limited laboratory tests such as urinalysis (19) and glucose (10). Seventeen clinics are able to administer IV fluids [13].

In 1989, Maccabi Health Services built the first UCC that was open 24 hours. This was a free standing unit that was dedicated to pediatrics. Subsequently, Maccabi opened general units that provided care until midnight and units that were dedicated to orthopedics [12]. Maccabi currently lists 9 UCC branches on its website that are open on nights and weekends. It is stated that they provide medical treatment but the specific services offered are not listed [14].

In the early 90s, the two remaining health plans, Meuhedet and Leumit followed suit [12], so that for the last two decades, all four health plans provide some form of urgent care. Meuhedet lists 25 evening clinics provided by the health plan [15]. Leumit lists over 90 urgent care clinics on its website but almost all of them are open morning and afternoon hours and not evenings and weekends [16]. All four health plans also allow use of at least some of the branches of the private UCC described below.

The first initiative to open a privately owned UCC in Israel began in 1988. A group of US trained physicians who had personal experience with UCC care in Cleveland, Ohio sought to open such a center in Jerusalem called TEREM (acronym for Immediate Medical Care in Hebrew). Their efforts were met with a lawsuit by the physicians who worked for the local Magen David Adom. The center was finally opened after 18 months in 1989. In 1993, a branch was added in Maale Adumim, in 1998, in Modiin, and, in 2002, in Beit Shemesh, where all three of these locations are small to mid-sized towns in the Jerusalem region. In 2005, a second Jerusalem branch was added in the southern part of Jerusalem. In 2012, a branch was opened in Bnei Brak, within the main population center of Israel, and another opened in September 2013 in Carmiel, which is situated in the north of the country. Most of these centers at first were open only evenings and weekends. However, at this point, the main branch in Jerusalem, the branch in Modiin and the branch in Bnei Brak are open 24/7/365. The opening of the branch in Bnei Brak was the first example in Israel of co-ownership with a hospital system as it represents a partnership of TEREM with the Tel Aviv Medical Center (Ichilov). TEREM clinics offer physician examinations and treatments such as casting and suturing. They also provide laboratory testing and radiologic services.

There are currently three other privately owned companies that run both UCC and physician home visit services. These are, in size order:

1) Bikur Rofe (Physician Visit) -This was founded in 1993 and is the company with the most clinics in the country (currently 30). These clinics are spread nationwide and are open evenings and weekends. Seven clinics have radiologic services and can apply casts. The rest have more limited laboratory services such as rapid strep testing, urine strip testing and EKGs [17].

2) Malram (acronym for Center for Immediate Medicine in Hebrew) -This company runs 5 clinics in the main population center of Israel (Ramat Gan, Kiryat Ono, Petach Tivka, Kfar Saba and Hadera). These centers, open evenings and weekends, can provide physical examination by a physician, electrocardiograms and prescriptions for medications. There are no radiological services and extremely limited laboratory services [18].

3) Marlam (Center for Immediate Medicine in a different Hebrew acronym order) – This company runs two clinics (Rechovot and Rishon LeTzion) that are open weekends and offer examinations by a physician [19].

The Ministry of Health has also recently begun a project to establish 12 UCC in remote locations. At the time of this writing, they exist in three southern towns - Arad, in conjunction with the city of Arad, Mitzpe Ramon, in conjunction with the Rashi Foundation and in Dimona, in conjunction with the Mirage Foundation. The city of Netivot also sponsors an urgent care clinic [20]. One of these UCC, just opened in July 2013 in Kiryat Gat, is operated by TEREM.

#### Free standing emergency departments

Another US model that started at about the same time as the FEC is the free standing emergency department (FED). While there is some variation among facilities, most FEDs offer both urgent and emergency care. Their radiological services are broader than the average UCC as they may include CT and ultrasound. They are staffed by emergency medicine physicians and nurses rather than family practice physicians and nurse practitioners. The majority, but not all, is open 24/7. Currently there is political pressure for them all to be open 24 hours per day. This move is part of a trend in which FEDs are attempting to distinguish themselves from UCCs by having a greater resemblance to hospital EDs [21]. In 1978, there were approximately 55 FEDs in the United States, although most of these facilities could have been classified as UCCs. The number of FEDs grew only slightly in subsequent years, until approximately the beginning of the current millennium. By the end of 2008, the American Hospital Association estimated that there were 191 hospital-affiliated FEDs, 31 FED that were owned and managed by physicians or other private groups (located mostly in Texas) and one FED that is not hospital-affiliated or physician-owned, located in Delaware [21].

Israel’s Magen David Adom runs a full FEC open 24 hours per day in Kiryat Shmona at the northern tip of Israel. In Jerusalem, TEREM has taken over the former emergency department of Bikur Cholim hospital. While this is presently run along the same UCC model as TEREM’s main branch (24/7 availability with provision of routine laboratory and radiological services), the facilities are present for provision of a broader range of laboratory tests and radiological services that may be further developed over time.

#### Walk in clinics

The goal of the first two models is to provide a subset of ED care. The walk in clinic, developed first in Canada, was designed to fill a different niche in the ED-physician office spectrum – faster access to primary care. This form of urgent care began in urban centers as an extension of physician’s offices that offered a range of services not normally found in the traditional family physician’s office. The key feature of these clinics, which began appearing in western Canada in the early 1980s, is that no appointment is necessary [22]. There has been some study as to the impact of these centers, but there is less information about their exact numbers or services [23,24]. A study of practices in Toronto determined that about 30 (out of 421 practices surveyed) met the definition of a walk-in center [25].

England’s National Health Service (NHS) developed its own form of walk-in clinic. An NHS walk-in center is characterized by extended opening hours and a convenient location, often within in a few minutes walk from a hospital. A key selling point is the ability to consult a health professional without the need for an appointment. This health professional is generally a nurse, supported by clinical assessment software [26]. In addition, the NHS sponsors a number of nurse-led minor injury units [27]. Forty two National Health Service walk-in centers opened in England in 2000–3, and a further 21 opened in 2004.

#### Retail clinics

The most recent arrival to the US urgent care scene is the retail or convenience clinic. These are small clinics located in retail stores such as pharmacy, discount or grocery chains. They handle a limited array of conditions that include both immunizations (particularly of the influenza and tetanus vaccines) and minor illnesses such as pharyngitis, rashes, and urinary tract infections. Patients pay a known, fixed price for care which is provided by a nurse practitioner or physician assistant. The first of such clinics began in the year 2000 [28]. This form of urgent care has proliferated in the last decade, with the number increasing ten-fold from 2006 to 2008 [29]. In 2009, almost 1,000 of these clinics existed in the United States [30]. Recently, some clinics are providing more expanded services for the management of chronic conditions such as hypertension, diabetes and hypercholesterolemia and preventive health services [31].

As seen from the descriptions above, different models have different emphases. These emphases can be organized into an analytic framework that is outlined in Table 1. Each row ranks the locations of care along the listed spectrum from lowest to highest. Temporal Convenience refers to hours of availability and Spatial Convenience relates to how likely a location of care is to be near the homes of the majority of people it cares for. This ranking is based on the assumption of patient use of one physician office or one local emergency department and the assumption of multiple branches of UCC and retail clinics. There are, of course, exceptions to these assumptions.

**Table 1:** Ranking of models along spectrum of impact in increasing order

	**Least**			**Most**
Services for comprehensive care	Retail clinic	Walk in clinic physician’s office	UCC	ED
Continuity of care	Retail clinic	ED UCC	Walk in clinic	Physician’s office
Temporal convenience	Physician office	Walk in clinic retail clinic	UCC	EC
Spatial convenience	ED Physician office	Walk in clinic	UCC	Retail clinic

|ED = Emergency Department, UCC = Urgent Care Center.

It can be seen from the table that community urgent care facilities are all developed to address the need for prompt care at reasonable cost. UCC is the model that is most likely to put the emphasis on provision of such care during the evening and night, sometimes at locations that are more accessible than hospital EDs or physicians offices (but sometimes at locations that require the same degree of travel). Retail centers, on the other hand, put the emphasis on provision of care in convenient locations, primarily during the day but with some temporal convenience if the store is open long hours. On-going care of both acute and chronic conditions is the main focus of the traditional physician’s office.

Due to the different emphases of the different models, there are likely to be differences in the populations that they serve. The first three models outlined above generally offer services that can be applicable to all age groups. As in the US UCCs are privately run enterprises, they can decide to limit their services to those sections of the population that they feel most comfortable treating. In Israel, TEREM treats all ages and conditions in all of its branches. Thus, for example, in 2013 to date, 3.5% of those treated were over the age of 90 and 40% were under the age of 18 (Zimmerman, unpublished data). A few of the UCC that provide services to the health plans, do limit their services by age group. The retail clinics, provide limited services and thus only assist those parts of the population who need those specific services.

As UCCs and retail clinics in the United States are generally NOT covered by health insurance, they are more likely to treat those who can afford to pay out of pocket. In countries which have government-supported health care insurance such as the UK and Israel, this is not likely to be a factor, as these services are covered and even encouraged.

### The impact of urgent care

There are four major arguments offered for the need for community-based urgent care - reduction of ED utilization and overcrowding, reduction of waiting time for outpatient physician appointments, reduction of costs, and patient convenience. In this section, we will analyze the actual impact of urgent care on these issues.

#### Reduction of ED overcrowding

Depending on the definition of urgency, at least 40% of visits to US hospital based EDs are for conditions that are non-urgent and likely to be able to be treated in other settings [32]. Similar data were obtained in an emergency department study in Israel. In this study, among the elderly, about 39% were non-urgent, among children it was 44% and among soldiers it was 53% [33].

It has been logically argued that if patients with such conditions could be treated elsewhere, such as urgent care settings, then crowding would be reduced [34]. However, at least in the United States, this has been shown to be a false assumption. Studies have shown that the main factor leading to emergency department overcrowding is that sick patients, evaluated by the emergency physician and admitted to the hospital, sometimes have no place else to go and remain in the emergency department. It is mainly a symptom of an overcrowded hospital, not the result of “inappropriate” emergency department use [35].

While this issue has yet to be explored in depth in other countries, anecdotally, this seems to be at least partially true in Israel as well. The patient flow “traffic jam” is compounded by the fact that many hospital admissions of ED patients are unnecessary [33].

In many countries, a large proportion of the patients who use urgent care are NOT those patients who would otherwise have gone to the ED but rather those who would have self-treated their condition. This is an additional reason that been offered for the finding of many studies that urgent care facilities, in general, do not impact on the numbers seen in local EDs [22,36-40].

In certain settings, UCCs DO make an impact on ED visits. One example is the case of minor injuries in the UK where the existence of minor injury units in the UK have been shown to reduce the use of hospital-based EDs [41]. While there are no official studies to date of the impact of all Israeli UCC, the TEREM system seems to have an impact on ED use, as suggested by the statistics of annual visits in the Jerusalem area for 2012. In that year, TEREM saw over 200,000 patients, almost equal to the numbers seen in all Jerusalem hospital EDs combined. (Solow A, Kovalsky Y, Kramer N, unpubished data, 2012). Over twice as many pediatric injuries in are treated in TEREM than in the largest hospital system ED in the city (Ivancovsky M, Zimmerman DR, Friedman E, Kovalski N, unpublished data). Countrywide, Israel Ministry of Health statistics show 35-40% less ED visits **per capita** in those areas of the country where a branch of TEREM is found [42].

It should be noted that even if a substantial proportion of patients who reach the ED could have been treated in the community, it might still be the case that the overwhelming majority of simple cases are nonetheless being handled in the community with the incorporation of UCC. It should also be noted that there is very little duplication between visits at TEREM and visits in the ED, as only 6% of patients are referred from TEREM to EDs (Zimmerman, unpublished data). Statistics from other UCCs are not readily available.

#### Reduction of waiting time for outpatient physician appointments

Another hope for urgent care was that it would reduce waiting times for appointments in physicians’ offices. In the United Kingdom (UK), research has shown that this is not the case [42]. One reason for not finding an impact may be related to duplication of care. In Canada, Bell and Szafran found that 46.2% of patients seen in a UCC were then seen again in their physician’s office within one week [43]. However, they did not differentiate between planned follow-up and actual duplication of care. No statistics as to the amount of duplicate care are currently available from Israel.

It should be noted that some use of urgent care is by physician referral. This generally occurs when the patients require services, beyond consultation with a physician, that are either not available in the office setting most hours of the day, such as laboratory testing, or services generally not available in that setting any hour of the day, such as suturing, casting, and intravenous fluids.

#### Reduction of cost

EDs are open at all hours of the day and are staffed and equipped to handle a wide spectrum of high acuity situations. The need to provide these services and constant availability means greater expenditures. These costs are divided among all visits leading to a higher fee per visit. If a community facility “specializes” in lower acuity urgent conditions, the costs can be easily lowered. In fact, this has been shown to be the case. Care in UCC has been shown to be significantly cheaper than that provided by EDs and the care in retail centers is even cheaper [5,30].

The cost of treatment of out-of-hours care in UCC in Israel has been shown to be significantly cheaper than emergency department visits. The full cost to of an ED visit for a patient without the health plan co-pay is 600 shekel. The full cost of a UCC visit without health plan subsidy is approximately 200 shekel (the exact cost varies by company) [44]. Therefore, all health plans recommend the use of UCC for out of hour care [13-16] and the National Health Insurance Law imposes significant fee on those whose self refer to the ED but much less to those who use UCC [45].

#### Patient convenience

Wanting care outside of working hours has been shown to be the main reason that people come to the ED with minor complaints [46,47]. The convenience of care during non-working hours has been shown to be a key reason why people choose a UCC over a personal physician [48,49]. To deal with cases that use the ED so as not to miss work or school, rather than cases that need hospital based care, some EDs have opened “fast tracks”. These are separate areas and/or specific staff that are dedicated to handle low acuity patients [50,51]. Fast tracks have been shown to provide speedier discharge that a traditional ED setting. However, as they simply represent re-allocation, rather than reduction of hospital based resources, they do not offer the cost savings of urgent care.

An interesting finding in Israel is that the issue of accessibility may be one of perception, rather than reality. In an Israeli study of elderly users of EDs in one area of the country, it was shown that 68% claimed they were using the ED because their clinic was closed and yet 90% actually were treated during hours when the clinics were open [Zohar AA, unpublished thesis]. This is finding is further strengthened by Canadian studies that show that most use of walk-in clinics is during hours that traditional offices are open [24,25].

As discussed above, convenience may be spatial as well as temporal. In more remote areas, UCCs may lessen the amount of time that one needs to travel to obtain care. This has been shown to be a factor in Canada [52] and is the impetus behind the Israeli Ministry of Health’s move to establish UCCs in the periphery.

### Response to the development of urgent care

The established medical community has generally responded to each new model of urgent care in each country with suspicion. One of the initial reactions in the United States was that having the word “emergency” in the title might lead people with conditions that could not be treated in these settings, such as acute myocardial infarction, to waste time in seeking care [53]. For this reason, many UCCs prefer to emphasize the word “urgent” rather than the word “emergency”. Many of their websites also clearly say something like “if this is a serious emergency, please call an ambulance or go to a hospital emergency department”. This may have also been one of the incentives for the National Association of Freestanding Emergency Centers (NAFEC) organization to change its name to the National Association For Ambulatory Care (NAFAC).

A second concern has been regarding quality of care. The truth is that there are little data available on the quality of the care that is delivered by any urgent care model. However, what data are available seem to indicate that similar quality is offered by UCCs, primary care physicians, retail clinics and the ED [54]. For example, one study showed no difference in two week revisit rate between retail clinics and physicians’ offices [40].

The retail clinic model has engendered particular concern as to its impact on the receipt of preventative care. However, a large study of this model in the state of Minnesota (at a chain that owned 52% of all the US-based retail clinics at the time) showed no difference between those who used these clinics and a control group in the amount of preventative care received from the initial visit through the subsequent three months [30]. To the degree that urgent care facilities offer immunizations, they may actually improve some preventive services.

In the US, many urgent care centers have tried to decrease the concern regarding interruption of continuity of care by assuring that there is communication with community physicians. In Israel, visits to a UCC run by a health plan are either currently incorporated (such as in Meuhedet) or in the process of computerization to incorporate (as in Clalit) the visits into the patients electronic medical record. Information on visits to non–health plan UCCs are not automatically available. TEREM has been a leader in the private sector with the development of the ROAM [*Rofe Amit Terem* – TEREM Doctor Colleague] system. In the ROAM system, physician sign on to obtain reports on their patient’s visits. Anytime a patient is seen in any of the TEREM branches and the patient identifies his/her primary physician, a link is sent by email within 24 hours to the noted physician if they are in the system. Through this link, the physician has access to the full visit report, any x-rays performed with their interpretation by a radiologist and any laboratory tests that were done (Zimmerman, unpublished data).

It may be that some of the critics of urgent care were motivated, at least in part, by fear of competition. On the one hand, these centers compete with office-based care due to their extended hours, leading to these clinics being received coolly at first by local medical practitioners. On the other hand, UCCs might compete with EDs and hospitals saw this as a form of unfair competition. In the United States, hospitals are required to obtain a Certificate of Need (CON) which entails a significant amount of bureaucracy. EDs are required to be open 24/7 and accept all comers regardless of ability to pay. UCCs, on the other hand, can decide on their hours of operation and these can be based on financial considerations [4]. UCCs can insist on payment in full prior to treatment.

The fear of competition may have been the motivation for the American Medical Association to lead a campaign to insist on UCC regulation. The founders of UCCs, however, argued that they are basically private physician offices and do not need to be regulated beyond what is expected in a physician’s office. Over time, the voices of the objectors have softened and most states currently do not insist on UCC regulation. As mentioned above, the opening of the first private UCC in Israel was held back by a lawsuit by competitors.

One of the counter responses to the issues raised above by those involved in the provision of urgent care has been an attempt to increase their professional recognition. Physicians and others in the United States interested in urgent care formed NAFEC in 1973 [55]. This trade organization developed standards of operation, relationships with government and insurance carriers and offered courses on how to set up such units. In 1984, the NAFEC changed it name to NAFAC and continues to function as a source of self administered practice standard certification.

Another urgent care professional organization is the American Academy of Urgent Care Medicine (AACUM). The AAUCM offers board certification to providers (as well as to centers) [56].

The most active professional organization is currently the Urgent Care Association of American (UCAOA). This organization offers external credentialing and publishes the Journal of Urgent Care Medicine and conducts bi-annual benchmarking surveys [57]. To date, UCAOA provides the main framework for a specialty of urgent care through its professional journal and annual conferences. It also sponsored the first urgent care fellowships – all of which are seen as advanced one year fellowships for those trained in family practice. At present, there exist three fellowship programs – one in the University of Nevada School of Medicine in Las Vegas, one in the University of Illinois College of Medicine in Rockford and one in the University Hospitals Urgent Care Network in Ohio. A fourth fellowship has been offered by a Staten Island Physician Practice in collaboration with JFK Family Medicine Department in New York City. However, as of this writing, this position has yet to be filled.

Much of the work to increase the academic recognition of urgent care will be continued by the Urgent Care College of Physicians (UCCOP). UCCOP was founded in 2012 as an outgrowth of UCAOA to further development of the physician specialty of urgent care in the United States. The goal of UCCOP is to standardize the fellowship training in the field, develop clinical tools such as practice guidelines and work toward the promotion of the specialty [58]. At present, the only country that recognizes urgent care as a distinct medical specialty is New Zealand [59].

Research in urgent care is starting to grow and Israel is making a significant international contribution, particularly in the research on the treatment of specific conditions in the urgent care setting. Since 2006, the TEREM system maintains a department of research and has published a number of articles [60-72], as well as presenting their research at both national and international meetings [73]. In the US, the Urgent Care Foundation was founded in 2010 to promote research activities to promote urgent care research [74]. An email survey of the three US-based post graduate fellowships which the author conducted revealed that no research projects have yet been done or published by participants in these programs at the time of the writing of this article. However, this is likely to change in the near future.

### Unique urgent care issues in Israel

In Israel, all UCC are currently staffed by physicians. This is in contrast to the situation in the United States and Canada where a combination of physicians and nurse practitioners is generally used and British walk-in clinics and US retail clinics where only nurses or other physician extenders are used. This reliance on physicians in Israel is due to the fact that the model of physician extenders does not exist in this country at this time. The appropriate use of physician extenders in conjunction with physicians is something that Israel stands to learn from other countries.

A small change has begun recently, in that certain hospitals have started to use medical students as “physician assistants.” TEREM has actually started a program to train nurses and paramedics to function as physician assistants specifically in their UCC. However, this is an in-house program that does not have formal academic recognition. The impact that these new trainees will have on the cost and quality of care is currently under study.

Another unique form of UCC in Israel is one whose impetus is religious rather than medical. While the provision of life-saving care on the Sabbath is permitted by Jewish law, some stringent observers of the law prefer to have an option to have care provided by those who are not Jewish (and thus not obligated in the Sabbath restrictions). To meet this need, a few cities and towns with large ultra religious populations (e.g. Netivot, Modiin Illit, Jerusalem, Betar and Bnei Brak) have UCCs that are open only on the Sabbath and Jewish holidays that are staffed by non-Jews.

A pressing need for Israeli policy makers is to further study UCCs in Israel. At present, this type of service is encouraged by the health system, but there is little analysis of the care that is given and its impact on health services and cost. Integration of data between the health plans, urgent care centers and hospitals would allow for more accurate information one which to base policy decisions. This type of integration would allow, for example, more in depth study of potential overlap of care which might prevent duplication of services and their attendant costs.

It is also important to more fully integrate the UCC services into the overall emergency medical services in the country. At present, there are three quite separate systems – hospital emergency departments, ambulance services and UCCs – with no organized method of transfer between them. The cost of ambulance transfer from UCC to hospital emergency department is not automatically covered by the health plans. This, at times, leads patients to refuse ambulance transfers even when clearly medically indicated such as in suspected acute myocardial infarction (Zimmerman, unpublished data). Overall re-organization of the system will likely need outside intervention, as each of these sub-systems are independently owned and operated.

## Conclusions

Community based urgent care facilities have become an important part of the medical landscape in a number of countries. However, they remain primarily on the fringe of organized medicine. Despite the important role of community-based acute care facilities in Israel, no nationwide study has been done in two decades. Studies of ED usage and misusage in Israel are incomplete without integration of data from the UCC sector. Health policy planning in Israel necessitates further study of urgent care use and its financial, organizational, patient service and clinical impacts.

## Abbreviations

ED: Hospital based emergency departments; UCC: Urgent care centers; FEC: Free-standing emergency center; FED: Free-standing emergency department; US: United States.

## Competing interests

The author works for TEREM Emergency Medical Centers as indicated below in Author Information. We feel that this association has lead to our interest in the field and is not a competing interest. However, we would be happy to hear the input of the editorial office on this issue.

## Authors’ contribution

DZ was the primary author of this paper. She was responsible for acquiring the data, and drafting the manuscript.

## Authors’ information

DZ is the Director of Research and Education at TEREM Emergency Medical Centers. She co-authored the first paper on quality of pediatric care in an urgent care setting with Dr David Applebaum, the founder of TEREM in 1992.
